# Effects of host genetics and environmental conditions on fecal microbiota composition of pigs

**DOI:** 10.1371/journal.pone.0201901

**Published:** 2018-08-07

**Authors:** Tereza Kubasova, Lenka Davidova-Gerzova, Vladimir Babak, Darina Cejkova, Lucile Montagne, Nathalie Le-Floc'h, Ivan Rychlik

**Affiliations:** 1 Veterinary Research Institute, Hudcova, Brno, Czech Republic; 2 PEGASE, Agrocampus Ouest, INRA, Saint-Gilles, France; Wageningen Universiteit, NETHERLANDS

## Abstract

Since microbiota may influence the physiology of its host including body weight increase, growth rate or feed intake, in this study we determined the microbiota composition in high or low residual feed intake (HRFI and LRFI) pig lines, of different age and/or subjected to sanitary stress by sequencing the V3/V4 variable region of 16S rRNA genes. *Allisonella*, *Megasphaera*, *Mitsuokella*, *Acidaminococcus* (all belonging to *Firmicutes*/class *Negativicutes*), *Lactobacillus*, *Faecalibacterium*, *Catenibacterium*, *Butyrivibrio*, *Erysipelotrichaceae*, *Holdemania*, *Olsenella* and *Collinsella* were more abundant in HRFI pigs. On the other hand, 26 genera including *Bacteroides*, *Clostridium sensu stricto*, *Oscillibacter*, *Paludibacter*, *Elusimicrobium*, *Bilophila*, *Pyramidobacter* and TM7 genera, and *Clostridium* XI and *Clostridium* XIVa clusters were more abundant in LRFI than HRFI pigs. Adaptation of microbiota to new diet after weaning was slower in LRFI than in HRFI pigs. Sanitary stress was of relatively minor influence on pig microbiota composition in both tested lines although abundance of *Helicobacter* increased in LRFI pigs subjected to stress. Selection for residual feed intake thus resulted in a selection of fecal microbiota of different composition. However, we cannot conclude whether residual feed intake was directly affected by different microbiota composition or whether the residual feed intake and microbiota composition are two independent consequences of yet unknown genetic traits differentially selected in the pigs of the two lines.

## Introduction

Gut microbiota influences the physiology of its host in many ways. One association between microbiota and a host is the influence of microbiota on body mass since obesity in rodents as well as humans has been associated with microbiota of a particular composition [1, 2]. Microbiota influence on a host’s body mass starts with the regulation of the host’s appetite [3]. Certain microbiota members degrade polysaccharide fibers which cannot be digested by the host and ferment them into low molecular weight products such as acetate, propionate or butyrate [4–6] thus providing the host with additional energy rich substrates. Of these, butyrate is the preferred energy source for colonocytes thus contributing to optimal colonocyte growth and efficient nutrient resorption [7]. Microbiota therefore has a considerable effect on a host’s appetite, feed intake and energy recovery, all affecting the final body weight. Not surprisingly, defined mixtures of bacteria are considered as probiotic supplements with a positive effect on performance in humans and different farm animals including pigs [8].

Feed intake and feed conversion are key parameters in the livestock industry. Within each population of farm animals it is possible to identify individuals with different feed intake and feed conversion characteristics [9]. In INRA, two lines of Large White pigs differing in their Residual Feed Intake (RFI), a measure of feed conversion, have been selected [10]. Briefly, RFI corresponds to the difference between observed feed intake and feed intake predicted by the growth rate and back fat thickness of the individual. High or low residual feed intake is heritable although genes or other factors responsible for the phenotype are yet to be identified [11–13]. Both lines also differ in behavior and energy metabolism [10, 14]. Since microbiota is suspected to influence appetite regulation [15] and feeding behavior [3], we were interested whether the selection for low or high residual feed intake (LRFI and HRFI) could have been associated also with the selection of microbiota of a particular composition. For that purpose, we determined the microbiota composition in LRFI and HRFI pigs in two independent experiments, in which the pigs differed in age. In addition, we also tested to what extent the weaning affects microbiota development in LRFI and HRFI pigs and if microbiota composition in both lines may change under poor housing conditions.

## Materials

### Ethical statement

The experiments were carried out in the experimental facilities of INRA Saint-Gilles (France). Animals were reared following French guidelines for animal care and use, and the experimental protocols were authorized by the French Ministry of Higher Education and Research (agreement APAFIS-2016010512258334 for the Experiment 1 and APAFIS#494–2015082717314985 for the Experiment 2).

### Animals

Large White pigs from the 8^th^ generation of a divergent genetic selection for residual feed intake were used in this study [12]. The lines were established using the residual feed intake selection criterion between 35 and 95 kg body weight, calculated as RFI = ADFI−(1.24 × ADG)−(31.9 × BFT), where ADFI was the average daily feed intake (g/day), ADG the average daily gain (g/day) and BFT was the back-fat thickness in mm at 95 kg [12]. Pigs of the LRFI line eat less than predicted and are more efficient whereas those from the HRFI line eat more than predicted and are therefore less efficient. For the present study, pigs were born in the INRA experimental facilities in Saint-Gilles (France) from 26 LRFI and 20 HRFI sows. For both experiments, irrespective of the lines and the experimental treatments, gestating and lactating sows and their litter, weaning and growing pigs were reared under the same conditions. This means that within the experimental treatments described hereafter, LRFI and HRFI pigs were housed in the same rooms and were fed the same feed.

### Experiment 1—Weaned piglets

**Fig 1:**
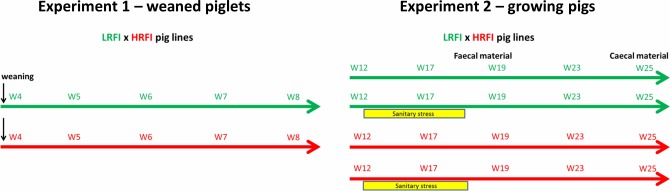
Experimental study design. Two independent experiments with pigs belonging to two different lines (LRFI and HRFI) were performed. The age of pigs at the time of sampling in weeks is indicated. In the experiment with growing pigs aged 12 to 25 weeks, half of the pigs were subjected to sanitary stress from week 12 to 18. Sampling on week 12 was performed just before the start of sanitary stress. Fecal material was collected from pigs from week 4 until week 23 while cecal contents were analyzed from pigs at week 25.

Seventy-two piglets housed in individual cages represented by an equal number of 36 piglets belonging to LRFI and HRFI lines were included in experiment 1. Piglets were fed successively two commercial diets (CCPA, Janzé, France): a starting diet for the first 11 days post weaning followed by the weaning diet until 10 weeks of age. The starter and weaning diets contained 18.9% and 16.6% of crude protein, 1.34% and 1.16% of lysine, and 12.3% and 15% of neutral detergent fiber, respectively. The diets were based on cereals (wheat and barley) and soybean meal. The starter diet also contained extruded soybean seeds, whey and bakery by-products. Fecal samples were collected from all piglets at weekly intervals starting from week 4 of life, just at the time of weaning, and ending on week 8 of life (Fig 1). Due to technical difficulties, the total number of successfully processed samples was 347, slightly less than 360 samples that would be expected for the given number of piglets and number of sampling periods. The missing samples were randomly distributed across all the groups and time points and did not affect any of the downstream analyses.

### Experiment 2—Growing pigs

Feces from 36 reared pigs, 20 from LRFI line and 16 from HRFI line, were collected on week 12, 17, 19, 23 and 25 of life. Diet for growing pigs consisted of 44.17% starch, 15.7% neutral detergent fiber, 5.6% acid detergent fiber, 1.6% acid detergent lignin and 3.14% fat supplied as wheat 32.2%, barley 30%, maize 15%, soya bean 7% and bran 5%. Half of the pigs of each line were subjected to sanitary stress lasting from 12 to 18 weeks of age. After this 6-week period, all pigs were transferred to the same clean room for the remaining 7 weeks prior to slaughter (Fig 1). The sanitary stress was achieved by housing pigs in rooms previously occupied by non-experimental pigs without any cleaning before and during the experiment. Conversely, half of the pigs were housed in clean conditions. To establish clean conditions, the room was cleaned and disinfected, in addition to the application of optimal aeration rate and temperature and strict biosecurity precautions. In both conditions, no antibiotic was systematically administrated. These two contrasted housing conditions are known to stimulate the immune system, to induce a systemic inflammatory response and to depress growth rate. The impact of this experimental model on performance and physiology of the pigs were fully described [11]. Briefly, prevalence of respiratory was higher in dirty conditions, including inflammation of lung tissue (pneumonia) or the surrounding membrane (pleurisy). The pig growth rate was on average 20% lower in dirty conditions and this reduction was greater for the HRFI line than the LRFI line (26 vs 12%). Poor hygiene conditions induced a systemic inflammatory response and oxidative stress, and this response was greater in HRFI pigs. Body weight at slaughter was lower for pigs that were reared under poor hygiene conditions (5.5 and 13.4 kg difference between clean and dirty for LRFI and HRFI, respectively).

Fecal samples were collected during the first four time points while cecal contents were collected from sacrificed animals on week 25. As in experiment 1, the total number of processed samples was 161, slightly less than 180 samples that would be expected for the given number of pigs and number of sampling periods.

### Microbiota characterization by next-gen sequencing of V3/V4 variable region of 16S rRNA genes

Fecal samples were homogenized using zirconia silica beads (BioSpec Products) in a MagNALyzer (Roche Diagnostics). Following homogenization, the DNA was extracted using the QIAamp DNA Stool Mini Kit according to the manufacturer’s instructions (Qiagen). The DNA concentration was determined spectrophotometrically and the DNA was stored at -20°C until use. PCR amplification over V3/V4 region of eubacterial 16S rRNA genes, DNA clean-up and MiSeq next-gen sequencing was performed as described previously [16]. The fastq files generated after next-gen sequencing were uploaded into Qiime software [17]. Quality trimming criteria were set to a value of 19 and no mismatch in the MID sequences was allowed. Reverse reads were shortened to a length of 250 bp and forward and reverse sequences were joined. In the next step, chimeric sequences were predicted by slayer algorithm and excluded from subsequent analysis. The resulting sequences were then classified by RDP Seqmatch with an OTU (operational taxonomic units) discrimination level set to 97% followed by UniFrac analysis. Principal coordinate analysis (PCoA) implemented in Qiime was used for data visualization. The raw sequence reads were deposited in the NCBI Short Read Archive under accession number SRP119641.

### Comparison of microbiota abundance in pigs from different lines, age or housing conditions

Read counts of individual OTUs in individual samples were converted to percentage representation and these were quantified at the genus or family level, as appropriate. Finally, we performed a Mann-Whitney test comparing the abundance of each family, genus or OTUs in samples originating from HRFI or LRFI pigs of the same age. In the second experiment, we also compared microbiota abundance in pigs of the same line and age but differing in their exposure to sanitary stress. Differences with p < 0.05 were considered as significant.

## Results

### Microbiota composition in LRFI and HRFI lines

**Fig 2:**
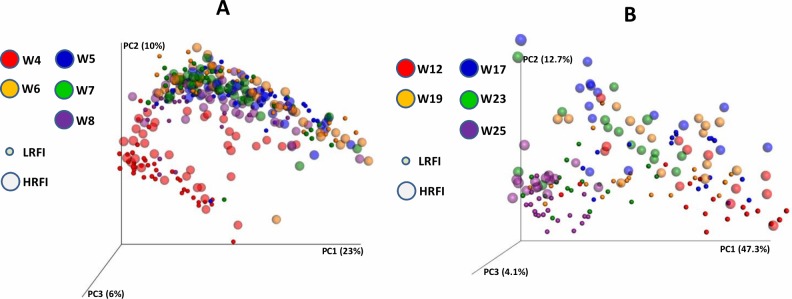
Microbiota composition visualized by weighted PCoA. Panel A, microbiota composition in piglets 4 to 8 weeks of age. Panel B, microbiota composition in pigs 12 to 25 weeks of age. Microbiota of pigs belonging to different genetic lines are differentiated by small or large symbols. Please note that the color scaling from the youngest to the oldest pigs follows the same pattern in both panels but the actual age of pigs in each panel is different.

Median sequencing coverage in the samples collected in the experiment with weaned piglets was 39,074 sequences per samples with minimal and maximal coverage 1,118 and 203,230 reads, respectively. Median sequencing coverage in the samples collected in the experiment with growing pigs was 14,413 sequences per samples with minimal and maximal coverage 3,579 and 30,304 reads, respectively. General analysis of microbiota composition visualized by PCoA showed an age-dependent development of gut microbiota followed by a moderate effect of pig line (Fig 2). These basal observations were confirmed also by indices characterizing population structure which showed that the complexity of pig fecal microbiota increased with age (S1 Table).

### Microbiota specific for LRFI and HRFI lines

**Fig 3:**
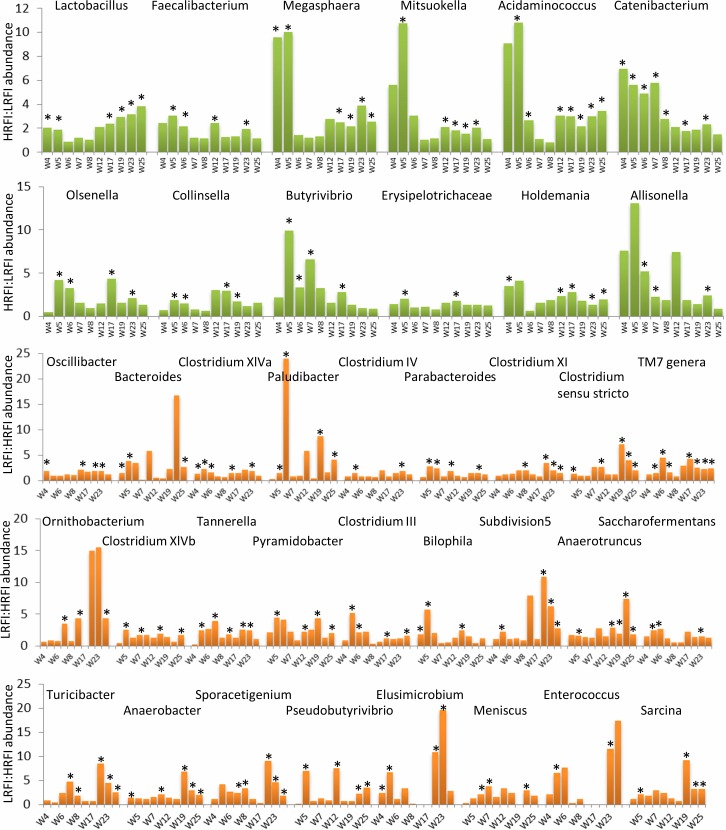
Differently abundant genera in microbiota of pigs belonging to HRFI and LRFI lines. Green columns indicate genera more abundant in the HRFI pig line. Orange columns indicate genera more abundant in the LRFI pig line. *—significantly different abundance in LRFI and HRFI pigs of a particular age, p<0.05.

Analysis of microbiota characteristic for each line was performed at the genus level. Selection criteria included a significant difference in the abundance between the two lines detected in at least one time point and in each of the two experiments, *i*.*e*. both in weaned piglets and growing pigs. In addition, we considered only genera which formed ≥ 0.05% of the total community in at least one pig. Thirty-eight genera passed these criteria (Fig 3). Twelve genera were more abundant in pigs from the HRFI than the LRFI line. These included genera *Allisonella*, *Megasphaera*, *Mitsuokella*, *Acidaminococcus*, *Lactobacillus*, *Faecalibacterium*, *Butyrivibrio*, *Catenibacterium*, *Erysipelotrichaceae*, *Holdemania*, *Olsenella* and *Collinsella*. All these genera belong to Gram positive bacteria of phyla *Firmicutes* or *Actinobacteria*. Within *Firmicutes*, orders *Selenomonadales* and *Erysipelotrichales* were represented by 4 or 3 genera, respectively. Twenty-six genera were more abundant in the LRFI pig line microbiota and these included *Bacteroides*, *Paludibacter*, *Parabacteroides*, *Tannerella*, *Meniscus*, *Ornithobacterium*, *Elusimicrobium*, *Enterococcus*, *Turicibacter*, *Clostridium sensu stricto*, *Anaerobacter*, *SarcinaPseudobutyrivibrio*, *Sporacetigenium*, *Oscillibacter*, *Anaerotruncus*, *Saccharofermentans*, *Bilophila*, *Pyramidobacter*, *Subdivision5 genera incertae sedis* and TM7 genera as well as *Clostridium* XIVa, *Clostridium* XIVb, *Clostridium* XI, *Clostridium* IV and *Clostridium* III clusters. These genera belonged to 7 different phyla of both Gram positive and Gram negative bacteria with no *Actinobacteria* or *Selenomonadales* representatives being more abundant in the LRFI than in the HRFI pigs.

### Microbiota development in LRFI and HRFI lines after weaning

**Fig 4:**
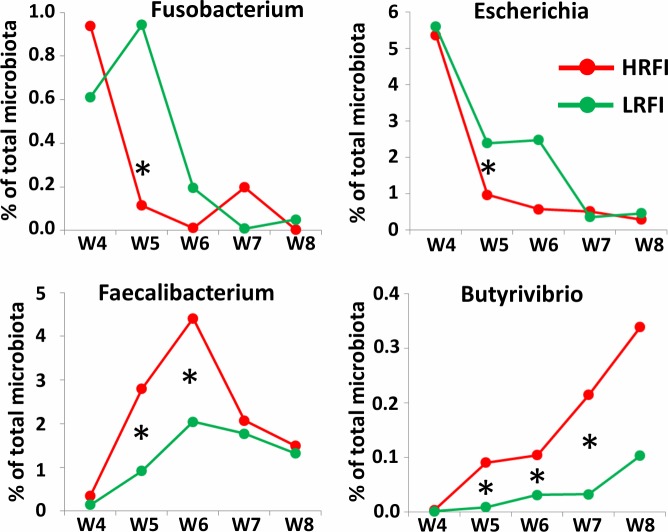
Four selected genera and their abundance in fecal microbiota of piglets after weaning. Differently abundant genera exhibited faster adaptation to new conditions after weaning in HRFI (red lines) than in LRFI piglets (green lines). * indicates significant difference in abundance between the two lines, p<0.05. Similar patterns were found for the other 30 genera with differential microbiota development in LRFI and HRFI pig lines after weaning.

Microbiota members differently selected in LRFI and HRFI lines after weaning were defined as those which were not differently abundant in the fecal microbiota of the two lineages at weaning (week 4 of life) but became significantly abundant one week later. There were 34 different genera which became differently abundant in fecal microbiota of HRFI and LRFI piglets after weaning. Of these, 12 were more abundant in microbiota of HRFI piglets (*Olsenella*, *Collinsella*, *Enterorhabdus*, *Butyricicoccus*, *Faecalibacterium*, *Butyrivibrio*, *Erysipelotrichaceae*, *Sharpea*, *Mitsuokella*, *Acidaminococcus*, *Asteroleplasma* and *Streptophyta*) and 22 were significantly more abundant in the microbiota of LRFI piglets (*Akkermansia*, Subdivision 5 genera, *Oxalobacter*, *Escherichia*, *Fusobacterium*, *Pyramidobacter*, TM7 genera, *Enterococcus*, *Sarcina*, *Howardella*, *Pseudobutyrivibrio*, *Fastidiosipila*, *Saccharofermentans*, *Hydrogenoanaerobacterium*, *Anaerotruncus*, *Pseudosphingobacterium*, *Parabacteroides* and *Paludibacter* as well as *Clostridium* XVIII, *Clostridium* III, *Clostridium* IV and *Clostridium* XIX clusters). Although this experiment was not repeated, the microbiota of LRFI piglets appeared more conserved while the microbiota in HRFI line piglets quickly adapted to new conditions after weaning (Fig 4).

### Microbiota members differently selected in LRFI and HRFI lines during sanitary stress

Abundance of 31 different genera significantly changed during sanitary stress in at least one of the pig lines, but only three of them, *Bifidobacterium*, *Streptococcus* and C*lostridium* XI cluster, responded to sanitary stress in pigs of both lines.

**Fig 5:**
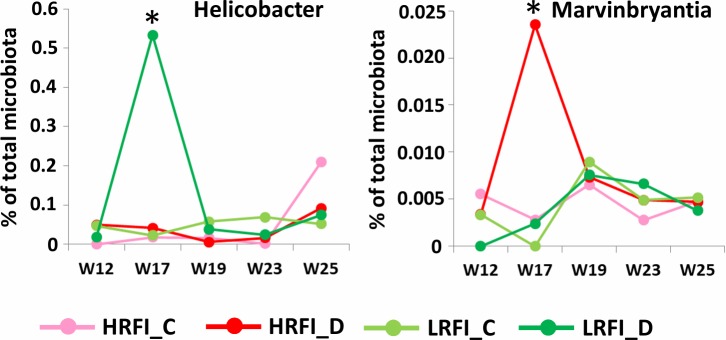
Bacterial genera differently selected in LRFI and HRFI lines during sanitary stress. *Helicobacter* increased only in microbiota of LRFI pigs and *Marvinbryantia* increased only in microbiota of HRFI pigs following sanitary stress exposure (“D” indicates Dirty, i.e. poor sanitary conditions and “C” indicates Clean, standard sanitary conditions). *—significantly different abundance in pigs subjected to sanitary stress in comparison to those kept under standard, clean conditions, p<0.05.

The abundance of 16 genera such as *Pilibacter*, *Lactovum*, *Fibrobacter*, *Prevotella*, *Faecalibacterium*, *Lachnospiracea incertae sedis*, *Saccharofermentans*, *Dialister*, *Lactobacillus*, *Eubacterium*, TM7 genera *incertae sedis*, *Phascolarctobacterium*, *Parasutterella*, *Helicobacter*, *Cellulosilyticum* and *Anaeroplasma* was affected by sanitary stress in LRFI pigs only. Except for *Pilibacter*, *Lactovum*, *Fibrobacter* and *Prevotella*, the abundance of all the remaining genera increased in microbiota of LRFI pigs subjected to sanitary stress. The abundance of 14 genera (*Streptococcus*, *Anaerobacter*, *Sarcina*, *Shuttleworthia*, *Enterorhabdus*, *Mucispirillum*, *Bifidobacterium*, *Clostridium sensu stricto*, *Paralactobacillus*, *Ruminobacter*, *Marvinbryantia* and *Sporacetigenium* as well as *Clostridium* III and *Clostridium* XI cluster) was affected by sanitary stress only in HRFI pigs. *Streptococcus* and *Clostridium* III cluster increased in the microbiota of HRFI pigs subjected to sanitary stress while the remaining genera decreased after exposing HRFI pigs to sanitary stress. However, when we individually evaluated the abundance of different genera in pigs subjected to sanitary stress, only *Helicobacter* and *Marvinbryantia* exhibited a profile clearly corresponding with the expected response to sanitary stress, *i*.*e*. low abundance before induction of sanitary stress, sharp peak in abundance during the sanitary stress and rapid decrease to abundance levels recorded before the stress. *Helicobacter* increased only in LRFI pigs while *Marvinbryantia* increased only in HRFI pigs following sanitary stress exposure (Fig 5).

## Discussion

In this study we analyzed the fecal microbiota composition in pigs of two lines differing in residual feed intake, from weaning until market age. Although there are reports that genetics of pigs might be of rather low consequences for gut microbiota composition [18], we recorded moderate differences in microbiota of HRFI and LRFI pigs reproduced in two different experiments. HRFI pigs were associated with bacterial genera which are commonly considered as beneficial such as *Lactobacillus*, *Faecalibacterium*, *Megasphaera*, *Olsenella*, *Collinsella* or *Butyrivibrio*. *Faecalibacterium*, *Megasphaera* and *Butyrivibrio* belong to butyrate-producing bacteria [5] with a beneficial effect on a host. We also noticed that the microbiota of pigs belonging to the HRFI line was enriched in *Actinobacteria* (*Olsenella* and *Collinsella*) and *Selenomonadales* (*Acidaminococcus*, *Allisonella*, *Mitsuokella* and *Megasphaera*). On the other hand, microbiota of the LRFI line was enriched in *Bacteroidales* (*Bacteroides*, *Paludibacter*, *Parabacteroides* and *Tannerella*) which are capable of fermenting complex polysaccharides including those produced by the host [4, 19, 20]. Microbiota of LRFI pigs developed more slowly than HRFI pigs during lactation (Fig 2A and compare small and large red spots with HRFI piglets clustering closer to older piglets) and also adapted more slowly than HRFI pigs to a post-weaning diet (Fig 4). Finally, sanitary stress did not result in extensive modification of fecal microbiota since only two genera exhibited a clear increase during the sanitary stress. However, one of them was *Helicobacter* which increased only in microbiota of LRFI pigs subjected to the sanitary stress. *Helicobacter suis* is usually associated with mucosal surfaces [21] and causes gastritis and leads to a reduction in body weight over time in experimentally infected pigs [22].

Although we cannot provide any conclusive explanation for all the recorded differences, we can exclude that vertical transmission of microbiota might be responsible for the different microbiota in the piglets of the two lines as the sows and piglets from the two lines were reared together. When gestating or lactating, sows from the two lines and their piglets were kept in the same room and were provided the same feed. At weaning, piglets from the two lines were combined. Herd separation as an explanation for the differences between the two lineages can therefore be excluded.

The faster adaption of microbiota from HFRI piglets towards microbiota characteristic for adult pigs may explain the lower incidence of post-weaning diarrhea in HRFI piglets than in LRFI piglets (4 vers 10 piglets with diarrhea during week 5 respectively, P<0.05), unpublished data). Abundance of *Fusobacterium* or *Escherichia*, which are common to microbiota of piglets under lactation [16, 18], decreased slowly in LRFI pigs after weaning and should pathogenic clones of *E*. *coli* be present among commensal *E*. *coli* strains, these would have a longer time window to cause post-weaning diarrhea [23]. Though stability of microbiota is an important factor with usually positive meaning, this need not be the case for post weaning adaptations when diet changes considerably and the piglets with faster adaptation to the new type diet may be more resistant to infections. Higher sensitivity of LRFI pigs to infections is consistent also with the increase of *Helicobacter* only in microbiota of LRFI pigs during sanitary stress. Phenotype of low or high residual feed intake can be partially affected also by the abundance of *Bacteroides* and *Parabacteroides*. Representatives of these genera can degrade and ferment complex polysaccharides into acetate and propionate [5, 24] which could be used by LRFI pigs as an additional energy source. This may explain lower feed intake in LRFI pigs necessary to reach the same body weight as HRFI pigs. On the other hand, the increased weight of digestive tract in HRFI pigs [13] could be caused by the presence of a higher amount of less efficiently digested fibers due to a lower abundance of *Bacteroides* and *Parabacteroides*.

Although we characterized differences in gut microbiota composition in HRFI and LRFI pigs, the fact that the pigs of both lines were co-housed in the same animal house shows that the pig was the decisive factor. If the conditions in the intestinal tract were the same in both lines, due to the co-housing, microbiota in both pig lines would converge to the same composition. It is likely that the different microbiota further contributes to the HRFI and LRFI phenotype due to the reasons discussed above. But we also cannot exclude the possibility that the different pig genetics is directly responsible for more efficient nutrient transport, irrespective of microbiota composition. In such a case, our observation of differences in gut microbiota of HRFI and LRFI pigs would represent only a correlation with the RFI phenotype but without any direct causation.

## Supporting information

S1 Table Indices characterizing population structure of porcine gut microbiota. (XLS)
